# Meat Quality Parameters and Sensory Properties of One High-Performing and Two Local Chicken Breeds Fed with *Vicia faba*

**DOI:** 10.3390/foods9081052

**Published:** 2020-08-04

**Authors:** Cynthia I. Escobedo del Bosque, Brianne A. Altmann, Marco Ciulu, Ingrid Halle, Simon Jansen, Tanja Nolte, Steffen Weigend, Daniel Mörlein

**Affiliations:** 1 Department of Agricultural Economics and Rural Development, University of Goettingen, 37073 Goettingen, Germany; 2 Department of Animal Sciences, University of Goettingen, 37075 Goettingen, Germany; brianne.altmann@agr.uni-goettingen.de (B.A.A.); marco.ciulu@uni-goettingen.de (M.C.); tanja.nolte@uni-goettingen.de (T.N.); daniel.moerlein@uni-goettingen.de (D.M.); 3 Institute of Animal Nutrition, Friedrich-Loeffler-Institut, 38116 Braunschweig, Germany; ingrid.halle@fli.de; 4 Institute of Farm Animal Genetics, Friedrich-Loeffler-Institut, 31535 Neustadt, Germany; simon.jansen@fli.de (S.J.); steffen.weigend@fli.de (S.W.); 5 Center for Integrated Breeding Research, University of Goettingen, 37075 Goettingen, Germany

**Keywords:** alternative protein source, Bresse Gauloise, chick culling, faba bean, fava bean, meat-type chicken, slow-growing, Vorwerkhuhn, White Rock

## Abstract

The current practices of the poultry industry have raised concerns among consumers. Among these is the culling of day-old male chicks of laying hybrids; a suitable alternative for this could be the use of dual-purpose breeds where both sexes are used. Another practice that causes concern is the import of large quantities of soybeans for feedstuff production. Substitutes for these soybean-based products are regional protein crops, such as faba beans (*Vicia faba* L.; FBs). The objective of this study was to test the suitability of FB as a locally produced soybean meal replacement for two local dual-purpose chicken breeds and one high-performing layer line. The breast and leg meat of male Bresse Gauloise (BG), Vorwerkhuhn (VH), and White Rock (WR) animals was evaluated for different meat quality parameters: pH, color, water holding capacity, and tenderness. Sensory properties of the samples were evaluated by a trained panel with a conventional descriptive analysis. Results show different effects of FB diets on meat quality parameters in the different breeds. The attributes mostly affected by the diet are related to aroma, flavor, and texture, particularly in VH and WR. Overall, faba beans appear to be an acceptable dietary protein source for rearing these breeds for meat production.

## 1. Introduction

Nowadays, commercial poultry breeding is characterized by specialized fattening (meat-type) and laying lines (egg-type), i.e., meat-type genotypes are not used for egg production and laying genotypes are managed for efficient egg production where carcasses of culled hens are considered a by-product. Contrary to meat-type genotypes, where both sexes are used, in laying hybrids, only hens are used for egg production. Since male offspring of layers do not produce enough meat, they are not used for fattening and are culled on their first day of life, in both organic and conventional farming. This practice has raised ethical concerns in some European Union (EU) countries, including Germany [1,2], leading to research into alternatives. One alternative to killing day-old male chicks is the use of dual-purpose breeds: breeds that produce meat (males) and lay eggs (females). As dual-purpose breeds have only become interesting in recent years, they are not able to keep up with specialized meat-type and laying breeds. Dual-purpose hens lay fewer eggs and the males produce less meat even when fattened over a longer period of time. These inefficiencies also mean an increase in production costs associated with feed and housing, resulting in higher product prices [3]. Yet, dual-purpose breeds could produce an improved meat quality and taste, in addition to meeting consumer animal welfare expectations; therefore, consumers might be willing to pay a higher price for these products [1,4,5].

The movement for dual-purpose breeds does not only stem from the ethical aspects surrounding the culling of day-old chicks. The current specialized lines of production have led to a limited gene pool used in poultry breeding; therefore, the use of dual-purpose breeds, particularly traditional (or local) breeds, is important to the conservation of poultry genetic resources [6,7]. Traditional breeds, such as the French Bresse Gauloise (BG) breed, have also been used in Germany as dual-purpose chickens, while local breeds, such as the Vorwerkhuhn (VH) originating from Germany, are mainly kept by hobby breeders. However, their laying performance is rather low. Crossbreeding of such traditional breeds with high-performing commercial laying hens such as White Rock (WR) could be used to produce a dual-purpose genotype with a higher laying performance [8].

Another problem in the poultry industry is that animal farming requires a high amount of protein-rich feedstuff. The production of these feedstuffs causes a greater environmental impact on the entire poultry farming system than rearing the animals [9]. Although the requirement for large amounts of protein-rich feedstuffs has attracted criticism in recent years, it will likely remain necessary to meet future demands for human dietary protein [10]. Soybeans, specifically soybean meal, are widely used as a protein source in poultry diet formulations; however, since the EU’s soybean yield is not sufficient to cover the requirements of its own poultry industry, there is a need to import soybean products from other countries, such as United States of America, Brazil and Argentina [11,12]. The large amount of soybean imports (13 million tons by the EU in 2016 [11] and 3.5 million tons by Germany in 2017 [13]) contributes to instability in the EU agricultural sector, mainly due to price volatility of soybeans on the global market and production sustainability issues [12]. Additionally, EU citizens are concerned with genetically modified soy crops and deforestation in the Americas [12,14]. Alternatives to soybean products as poultry feed ingredients are regionally grown protein crops, such as beans and peas. These would contribute to a greater independency of local agricultural industries, as they would no longer have to rely on soy imports and their volatile prices, and provide environmental benefits like biological nitrogen fixation, in addition to having the potential to increase poultry production efficiencies [15].

Faba beans (*Vicia faba* L.; FBs) are one of the oldest and most widely cultivated legumes [16]. They contain approximately 30% protein [17], which is complemented further by an advantageous amino acid composition rich in lysine, yet variable in methionine and cysteine [16]. These characteristics make the FB a suitable candidate as poultry feed protein source [17]. In spite of their high nutritional value, FBs are considered to contain antinutritional factors (i.e., vicin and convicin; together abbreviated as VC) that have challenged their use in poultry diets [18,19]. The levels of VC vary depending on the FB cultivar [16]. It remains unclear whether a modern low-VC cultivar contains a low enough amount of antinutritional factors to allow for the substitution of soybean meal with FB in a poultry diet [20]. Literature is conflicted regarding the effect of FB as a dietary protein source in poultry diets. Not all findings are conclusive and appear to depend on the antinutritional characteristics of FB as well as bird physiological development [17]. For example, Laudadio et al. [21] and Dänner et al. [20] find that FB can be included in laying hens’ diets without having a significant negative effect on laying performance or egg quality; however, it was found in [22] that the inclusion of FB in laying hens’ diets can decrease egg weight. In addition, broiler nutrition (apparent metabolizable energy; AMEn) values are found to be adversely affected by antinutritional factors in FB; however, adult cockerels appear to be more resilient towards antinutritional factors [17].

The main objective of this study was to test the effect of FB as a locally produced soybean meal replacement on meat quality traits, including sensory analysis, of cockerels for two local dual-purpose chicken breeds and one high-performing laying line. The locally grown FBs contain average and reduced VC contents in order to ascertain the limit of antinutritional factors in these genotypes. Furthermore, this system offers the chance to fatten the brothers of laying hens of local breeds in a regional production system and therefore to refrain from culling male chicks. More specifically, the aim of this study is to assess whether traditional breeds can be used as a basis to develop a local alternative poultry production system based on their meat quality and sensory characteristics under different diets. The effect of poultry diets containing different local FB cultivars in these particular genotypes remains the variable under investigation.

## 2. Materials and Methods

This experiment is in accordance with the European Union directive on the protection of animals used for scientific purposes (Directive 2010/63/EU) and was approved by the Lower Saxony State Office for Consumer Protection and Food Safety (LAVES; ref. 33.9-42502-04-17/2622).

### 2.1. Animal Management and Sampling

One-day-old male chicks of Bresse Gauloise (BG), Vorwerkhuhn (VH), and White Rock (WR) breeds were reared in indoor pens using a commercial starter at the Friedrich-Loeffler-Institute (FLI; Celle, Germany) for three weeks. The BG and VH chicks were directly hatched at FLI; WR chicks were provided by from Lohmann Tierzucht GmbH (Cuxhaven, Germany). At 21 days, 120 BG, 94 VH, and 120 WR male chicks were transported to the Department of Animal Sciences at the University of Goettingen (Goettingen, Germany), where 40 chicks of each genotype were randomly assigned to one of three feed groups. Decreased hatchability of VH chicks resulted in reduced feed group size (approx. 30 animals per feed group). In total, there were nine different experimental groups (3 breeds × 3 feed groups). The chickens were reared in an indoor-floor system with a solid floor and with fans for ventilation and cooling. The density of each pen was 10 birds/3 m^2^, with the exception of VH, where seven to eight birds were held per pen; for each feed/breed combination there were four replicates. The temperature was held constant at 20 ± 2 °C, and the photoperiod was 16 h.

Three different diets (Table 1) were fed across all breeds starting at day 21. The control (C) group was fed soybean-meal-based feed, while the rations of the other two groups were based on FB feed mixture. The difference between the two FB-based diets was the VC content: one diet had a high (0.14%) VC content (VC+); while the VC content of the other diet was low (0.02%) (VC-). Table 1 outlines the ingredient composition of each experimental diet in percentage of ingredient per kg of feed. All animals were provided feed (pelleted) and water ad libitum.

The animals were reared from September 2017 to December 2017 or January 2018, therefore reaching slaughter ages of 10, 15, and 16 weeks for BG, VH, and WR, respectively. Age differences are due to different growth rates of the breeds reaching the same body weight (approximately 2100 g) at slaughter. At slaughter, the birds were electrically stunned, exsanguinated by neck cut, scalded, eviscerated, weighed, and chilled at 4 °C for 24 h. Twenty-four hours after slaughter (post mortem; p.m.) the carcasses were weighed and manually dissected. Results concerning the animals’ growth performance and carcass parameters have been reported elsewhere [23].

For each of the nine groups (3 breeds × 3 feeds), approximately 20 animals (BG 20, VH 16, WR 20) were allocated for sensory analysis, and 10 samples per group were used for physicochemical analyses; the rest of the samples were used for analyses not pertaining this study.

### 2.2. Physicochemical Analysis

The following physicochemical analyses were conducted on 10 samples per feed/breed combination: pH; color; water holding capacity (WHC), measured as storage loss and cooking loss; instrumental tenderness, measured as shear force; and content of flavor-related nucleotides, i.e., inosine-5′-monophosphate (IMP), adenosine-5′-monophosphate (AMP), and inosine. pH and nucleotides were analyzed in the left breast. Remaining parameters were recorded using the right breast samples, which were stored between 24 and 72 h p.m. in modified atmosphere (80% O_2_/20% CO_2_) packaging (MAP) using a PP tray with absorbent liners and heat-sealed with an oriented OPET/PP film (<3 cm^3^/m^2^/24 h bar oxygen transmission rate; <12 cm^3^/m^2^/24 h bar carbon dioxide transmission rate) using a vacuum packaging machine (TS 100, KOMET Maschinenfabrik GmbH, Plochingen, Germany) and stored at 4 °C without illumination. The pH values were determined at three different times (20 min p.m., 24 h p.m., and 72 h p.m.) by inserting a pH-electrode and a thermometer (Portamess 911, Knick Elektronische Messgeräte GmbH & Co. KG, Berlin, Germany) into the cranial part of the left breast. The pH-meter was regularly calibrated between breeds, using standard buffers for pH 4 and pH 7 at room temperature. Color was quantified using CIELAB coordinates (L*a*b* values). Three measurements were taken on non-overlapping areas (free of obvious color defects) using a colorimeter (CR-600d, Konica Minolta, Tokyo, Japan). Color was recorded on the ventral part of the right breast with skin at 24 h p.m. and without skin at 24 and 72 h p.m. The average of the three-color measurements was used in further analysis. The spectrometer was calibrated before every session using a white tile provided by the manufacturer. Storage loss was monitored by weighing the right breast at 24 h p.m. prior to packaging and reweighing at 72 h p.m., where the percent difference in weight was attributed to storage loss. Afterwards, these samples were frozen at −20 °C until cooking loss and shear force analyses were conducted (approx. 8 weeks p.m.). Samples were thawed overnight at 4 °C and freshly vacuum-packaged; then they were immersed in a hot water bath (1092, GFL Gesellschaft für Labortechnik mbH, Burgwedel, Germany) at 80 °C for 50 min until reaching a core temperature of 76 °C, as measured by inserting a thermometer (926, Testo SE & Co. KGaA, Lenzkirch, Germany). After cooling to room temperature, the samples were weighed in order to calculate the cooking loss as a percentage of overall weight loss. The samples were later wrapped in aluminum foil and stored overnight at 4 °C. Prior to conducting shear force analysis, samples were left unwrapped at room temperature for 10 min. Shear force values were measured with a TA.XTplus Texture Analyzer (Stable Micro Systems, Surrey, UK) equipped with a 5 kg load cell and a Meullenet-Owens Razor Shear Blade (MORS-Blade). The conditions for the test were the following: pretest speed 2 mm/s, test speed 10 mm/s, trigger type 10 g. Each breast sample was sheared four times perpendicular to the muscle fiber orientation, with a 1.5 cm distance from each cut. Results of each sample are presented as an average of the four measurements. Shear force is reported as the peak shear force (N) which is necessary to completely shear through the sample.

To determine the content of IMP, AMP, and inosine, samples of raw meat from the left breasts (5 samples per group) and left legs (10 samples per group) were taken at 24 h p.m., frozen with liquid nitrogen, and stored at −72 °C. Six months after slaughter, IMP, AMP, and inosine content was determined using the method of Morzel and Van De Vis [24] with some modifications. Minced samples (0.200 g) were homogenized (Schuett-homgenplus homogenizer, Schuett-biotec GmbH, Germany) with 1 mL of 5% (*w/v*) TCA (aq) for 1 min at 1600 rpm (Pico & Fresco 17/21 centrifuge, ThermoElectron LED GmbH, Osterode, NE, Germany) followed by chilling on ice for 15 min. The liquid extract was centrifuged at 4 °C for 5 min at 12,000 × *g*. The supernatant (200 µL) was diluted 1:4 (*v/v*) for the breast samples and 1:2 (*v/v*) for the thighs, with 5 % (*w/v*) TCA (aq) at pH 7.0. Extracts were kept at −20 °C before being injected into the HPLC system. The system (VWR Hitachi, Chromaster) was equipped with a 5260 pump, a 5260 autosampler (injection volume: 10 µL), and a 5410 UV detector operating at 260 nm. A LiChroCart Licrosphere 100 RP8 (250 × 4.6 mm, 5 μm) column was maintained at 30 °C in a 5310 column oven. The mobile phase consisted of 100 mM KH_2_PO_4_ (aq), 1.44 mM TBAHS (aq), and 0.5% methanol (aq, pH 7.0). Quantification was performed by an external calibration method. Identification of the analytes was performed by comparison of retention times. All analyses were performed in duplicate.

### 2.3. Sensory Analysis

**Figure 1:**
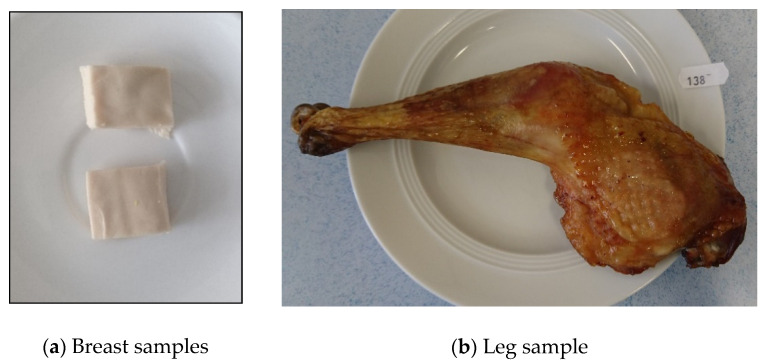
Pictures of (**a**) breast and (**b**) leg samples prepared for sensory evaluation.

The samples for sensory analysis were stored between 24 and 72 h p.m. in MAP packaging under the same conditions as samples for physicochemical analysis (see Section 2.2). At 72 h p.m., the samples were vacuum-packed in plastic bags and frozen at −20 °C until training or evaluation. All training and evaluation sessions took place in the sensory laboratory at the University of Goettingen, which complies with the international standard ISO 8589. All samples were thawed overnight at 4 °C prior to cooking for training or evaluation. Chicken breast samples were prepared according to the cooking loss procedure (see Section 2.2). The breasts were cut in 1 cm^2^ pieces and served on warm plates (Figure 1a) marked with a 3-digit code. Leg samples with skin were roasted in a commercial oven for 35 min at 190 °C and 50% air humidity until they reached a minimal core temperature of 76 °C, measured by inserting a thermometer (926, Testo SE & Co. KGaA, Lenzkirch, Germany) into the thigh, and kept warm in a food warmer (Bain Marie, Bartscher, Salzkotten, Germany) until served. Each panelist received one complete leg on a warm plate marked with a 3-digit code (Figure 1b).

Conventional descriptive analysis was carried out by a trained panel consisting of 10 assessors, who were experienced in descriptive sensory profiling of meat-related products and were trained and selected according to ISO 8586. All assessors provided written informed consent prior to participation.

The panel evaluated different chicken breasts and chicken legs from three breeds (BG, VH, WR) fed with three different diets (C, VC+, VC-), resulting in nine different products per cut (breast, leg). Assessors defined attributes in appearance, odor, taste, flavor, and texture that best described the samples and were trained further in these. To evaluate the breast samples, a total of 21 attributes were collected. Similarly, a total of 20 attributes were collected to describe the leg samples. A list of all attributes along with their definitions and scales for breasts and legs are presented in Table A1 and Table A2 (Appendix A), respectively. Training per cut was directly followed by evaluation of the nine products per cut, i.e., chicken breast training and evaluation was completed prior to starting sensory analysis of leg products.

The trained panelists evaluated the nine chicken breast products in triplicate in four sessions, where each assessor evaluated six samples per session. Leg products were only assessed in duplicate. All samples were evaluated, in a sequential monadic manner, in three different set orders that were allocated to three or four assessors for each session. After the evaluation of each sample, panelists were asked to neutralize their senses by drinking water; additionally, untoasted white bread was available for neutralization. Using EyeQuestion survey software (Version 4.8.7, EyeQuestion, Elst, the Netherlands), each sensory attribute was evaluated on a 10 cm unstructured line with an unmarked scale ranging from 0 (no perception) to 100 (strong perception). The electronically collected data were later used for statistical analysis.

### 2.4. Statistical Analysis

Due to the different slaughter ages, the statistical analyses of the evaluated physicochemical and sensory data were done separately for each breed; therefore, the feed effect was compared within breed.

Data analysis of physicochemical parameters was performed with SPSS (Version 24, IBM Corporation, New York, NY, USA) statistical software. Mean values were calculated and feed effect was compared within each breed with a one-way ANOVA using Tukey’s multiple comparison statistical test at a 95% confidence level (α = 0.05).

For the statistical analysis of sensory data, the linear mixed model (LMM) procedure from SPSS (Version 26, IBM Corporation, New York, NY, USA) was used for mixed model calculations. All calculations were compared within each breed. In the statistical model for breast samples, “feed” was defined as a fixed effect, while “panelist”, “animal”, “feed*panelist”, and “feed*animal” were defined as random effects. In the statistical model for leg samples, “feed” was defined as a fixed effect, while “panelist” and “feed*panelist” were defined as random effects. Within the model, a least significant difference (LSD) statistical test at a 95% confidence level (α = 0.05) was used.

For the sole purpose of visualization, a principle component analysis (PCA) was carried out with treatment group means across all parameters. The PCA was computed using RStudio (version 1.2.5033, R Foundation for Statistical Computing, Vienna, Austria) coupled with the FactoMineR package [25]. Variables were standardized and number of components was assessed based on a scree-plot.

## 3. Results

### 3.1. Physicochemical Results

When evaluating physicochemical parameters, the feed had an apparent effect on chicken breast quality depending on breed (Table 2 and Table 3). Across feed groups, no significant effects were observed in VH samples. pH values in both BG and WR with VC+ diets showed significant differences vs. control. While a VC+ diet significantly decreased the pH for BG at 24 h p.m. vs. control, the effect was the opposite in WR. In WR, the inclusion of VC increased the pH value of the samples at 24 and 72 h p.m. when compared to C; this increased pH value was significantly higher than that of C when WR was fed with a VC+ diet.

The main differences in BG were found in the color of the samples, mainly in the redness (a*) of the meat. For samples measured at 24 and 72 h p.m., a diet with FB significantly increased redness of the chicken breasts without skin (meat color) in only BG birds compared to a diet with soybean meal; although only VC+ and C are statistically significant, VC- values remained increased as compared to C for both BG and VH breeds. However, vicin content (VC- vs. VC+) did not statistically affect meat color. In WR samples, VC+ led to a significant decrease in yellowness (b*) of meat color at 24 and 72 h p.m. when compared to C. This change was also present in the skin tone of the samples at 24 h p.m. Overall, the color trend in WR samples was that an increasing vicin content resulted in less yellow tones of skin and meat. Furthermore, at 24 h p.m., a VC- diet significantly increased the lightness (L*) of samples when compared to a VC+ diet; this effect was seen in both BG and WR breeds.

Aside from differences in color, WR also showed significant differences between the different diets in storage and cooking loss. In contrast to storage loss, where C samples lost more moisture than VC+ and VC- samples, the faba bean diets resulted in a higher loss of water in cooking loss. Feed also had a statistically significant effect on shear force in WR birds; control samples of WR showed a significantly higher shear force when compared to WR chicken breast produced using faba bean (VC+ and VC-) diets.

IMP resulted as the most abundant nucleotide for both tissues and for all the genotype/feed combinations, followed by inosine and AMP. Breast samples showed no significant difference between feed groups within the same breed. In the case of thigh samples, instead, a significant difference (*p* < 0.05) between the control and the VC- group was found for the IMP content of the WR chickens.

### 3.2. Sensory Results

Across the three breeds, feed appeared to play a limited role in influencing organoleptic quality. Table 4 outlines the sensory attributes that were affected in at least one breed by feed in the breast samples; results for all sensory attributes can be found in the supplementary material (Tables S1–S3).

Results of BG breast filets showed only a difference in the fibrousness, measured as the degree of visible fibers on the cut side of the sample. BG chicken breast samples produced with a VC- diet had a more fibrous appearance than samples with a VC+ diet. For VH chicken breast, barn aroma and tenderness had statistically different values dependent on feed. The VH samples produced with VC- had a more reduced barn aroma than VC+ samples, yet with no significant difference to C samples. Likewise, tenderness differed between VH chicken breasts produced with VC- and VC+, where the lower vicin faba bean feed (VC-) contributed to a more tender product. Finally, tenderness was also influenced by feed in the production of WR chicken breast. Tenderness was significantly higher for feed group C compared to the VC- group, but with no difference to a VC+ diet.

Similar to chicken breast samples, only a few sensory attributes were affected by feed in a roasted thigh and drumstick (leg) product. Table 5 presents the sensory attributes that showed statistically significant difference in at least one breed by feed in the leg samples; results for all sensory attributes can be found in the supplementary material (Tables S4–S6). Feed played a larger role in the organoleptic quality of VH legs, whereas BG and WR samples remained mostly unaltered. Faba bean feed tended to increase the crispiness in BG leg samples; samples produced with VC- had significantly higher crispiness compared to the control group. Feed affected aroma, flavor, and texture attributes in VH legs. A high vicin content (VC+) significantly increased the barn aroma when compared to control; however, a low vicin content had no significant difference to samples fed with control. The control feed resulted in a product that tasted more metallic and had a more intensive aftertaste, overall. The high-vicin faba bean feed had significantly lower values for metallic flavor compared to the control group; the low-vicin feed resulted in a reduced aftertaste. Furthermore, samples of animals fed with VC- were significantly juicier than those of the VC+ diet; however, they were no different than the C group. Finally, flavor and texture attributes in WR were affected by the different diet groups. Animals fed with a VC- diet had a significantly less greasy/oily flavor when compared to control. Similarly, a faba bean diet decreased the crispiness of the samples, particularly for the VC- diet, when compared to control feed.

### 3.3. Overview of Interaction between Physicochemical and Sensory Characteristics

**Figure 2:**
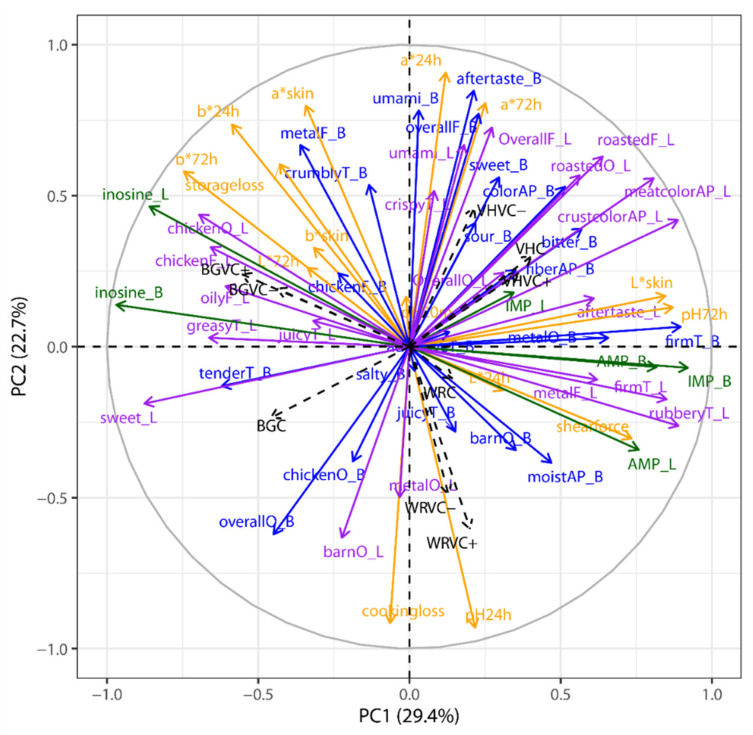
PCA loading plot showing the correlation of all physicochemical meat quality parameters, sensory variables, and nucleotide levels. Per group (breed type × feed type, *n* = 9) arithmetic means were used and standardized across groups such that correlations instead of co-variance are used for the PCA.

An overview in the form of a principle component analysis of group means is presented in Figure 2. Here, it becomes obvious that breed (associated with age of slaughter, etc.) plays an important role in characterizing quality, but it is also apparent that FB affects meat quality, whether physicochemical or organoleptic. Vicin and convicin content of the faba bean plays a limited role in WR and BG breeds, where VC+ and VC- groups congregate together. However, the same cannot be said for VH, where VC+ and C group together.

## 4. Discussion

Our study illustrates that, as within other more specialized poultry production systems (i.e., meat-type and laying), faba beans present themselves as a feasible dietary protein source. Although total product yield is an important factor to consider with different feedstuff groups, it is not the only defining factor in evaluating the acceptability of FB in poultry meat production. Therefore, our study focused on evaluating the effect different FB diets have on physicochemical as well as organoleptic meat quality of three different breeds; few previous studies focus on such aspects.

pH is one of the most important physicochemical characteristics in meat since it is related to water holding capacity and color. Similar values to BG and VH for pH at 24 h p.m. were obtained by Siekmann et al. [26] for a dual-purpose hybrid (Lohmann Dual) and by Muth et al. [27] for BG. Although there are slight differences in pH values between the different breeds (which might be attributed to other factors such as age or genetics), within each breed there has been no effect between feed groups in pH at 20 min and 24 h p.m. This is also reported in [19,28], where researchers do not report any significant differences between soy-based and faba-bean-based diets in chicken breast. Nonetheless, we observe that a high content of VC caused a low pH value at 72 h p.m. in BG. 

Faba beans were found to influence meat color in broiler chickens by Laudadio et al. [19] and in guinea fowl broilers by Tufarelli and Laudadio [28]. This is important given that meat color is assumed to be one of the most important characteristics evaluated at the point of purchase [29]. An unfamiliar product color can negatively impact consumer expectations [30]. In our study, FB diets did not affect color uniformly across breeds. In WR, b* values were significantly reduced with FB diets with or without skin and in both 24 and 72 h p.m. chicken breast. No color differences were recorded in other color parameters or in other breeds; therefore, it is likely that the color difference in WR chicken breast skin is due to the refraction of the altered lean meat color (below the partially translucent skin). The lower b* values are contradictory to the findings of Laudadio et al. [19], who fed a wheat-based diet where the control group dietary protein source was soybean meal and the test group dietary protein source was faba beans. WR chicken breast samples are also lighter (L*) in color when birds are fed a VC- diet; however, FB in general did not increase lightness. Laudadio et al. [19] observed darker samples with the feeding of faba beans in broiler chicks, whereas Tufarelli and Laudadio [28] found faba beans to increase lightness in guinea fowl broiler meat. In BG chicken breasts, increased redness values (a*) are observed for both time periods (24 and 72 h p.m.); this corresponds with the findings of [19] in broiler chicken meat. We do not observe an effect of diet on product color in VH samples, illustrating the need to not overgeneralize the effect of feed on meat color across breeds.

We also observe an effect of FB feed on instrumental tenderness and water holding capacity, where WR chicken breast samples raised on FB diets have increased water holding capacity (reduced storage losses) and are more tender (decreased shear force) as measured by a texture analyzer. Laudadio et al. [19] also observed a statistically significant increase in water holding capacity (as measured by a filter paper–oven drying method) in their faba-bean-fed broiler chicken breast samples; drip loss also tended to be decreased (as measured with the filter paper method). Tufarelli and Laudadio [28] also verified faba beans’ effect on water holding capacity in guinea fowl broilers. Unfortunately, to the best of our knowledge, no studies exist investigating instrumental tenderness of poultry meat from chickens fed faba bean diets. However, it would be expected that instrumental tenderness values are reduced (i.e., samples are more tender) with an increased water holding capacity.

AMP, IMP, and inosine all derive from the breakdown of adenosine triphosphate (ATP), which occurs in muscle during the slaughter and the postmortem aging phases [31]. Among these, IMP plays a predominant role in the formation of meat flavor by contributing to the umami taste [32]. The data obtained in this study confirm that IMP is the most abundant nucleotide, while AMP shows the lowest concentration values [33,34,35]. Furthermore, across all the three breeds in this study, IMP and inosine contents tend to be higher in chicken breast compared to the legs, as already observed in other studies [33,34,36].

With regard to the effect of the feed for each of the genotypes, from a general point of view it seems that the replacement of soybean meal with FB has no significant effect on the content of the selected nucleotides. In the past, levels of IMP in chicken meat were proved to be related to dietary purine nucleotide, betaine, and soybean isoflavone contents [37]. Although there is a lack of information about the concentration of these compounds in the three experimental diets, we can assume that the replacement of soybean meal with FB did not lead to significant changes in their levels.

To the best of our knowledge, we are the first to investigate the effect of faba bean diets on organoleptic properties of poultry meat. Furthermore, in profiling three different breeds, our results offer three distinct sensory profiles for Bresse Gauloise (BG), Vorwerkhuhn (VH), and White Rock (WR) poultry meat; to the best of our knowledge, these have also not yet been documented. Instead, research has focused on creating sensory profiles of meat from chickens reared in different production systems (e.g., [4,38,39,40]) or compared to other indigenous breeds (e.g., [41,42]) or to other specific diets (e.g., [38,43]).

As research has shown, depending on several factors like breed [44], feedstuff [38], and age [4,39], chicken breast samples have different attributes in different intensities that best describe their organoleptic properties. However, there are a few general attributes that are present in chicken meat despite the abovementioned factors, such as chicken and metallic aroma, umami taste, and chicken and metallic flavor. Texture is one of the most important sensory qualities associated with consumers’ satisfaction [45], and attributes such as firmness, tenderness, and crumbliness are usually of interest for meat samples. Horsted et al. [39] showed that chicken aroma, chicken flavor, and umami taste in breast samples are positively correlated to the product’s overall liking, while a metallic aroma and taste have a negative correlation to the overall liking. A study by Lawlor et al. [38] showed that for one of five groups of consumers, firmness of the initial bite was correlated to product preference, while for a different group an astringent taste and a crumbly texture were positively correlated to overall liking. On the other hand, for roasted chicken samples, Sow and Grognet [46] showed that juiciness, oiliness, sweetness, and hardness are attributes correlated to product preference, while chewiness, astringency, and smoothness were negatively correlated to preference.

In general, the effect of the different feed groups led to slight organoleptic changes in BG breast and leg samples. Breast samples of animals fed with FB showed a slight, not statistically significant, improvement in their sensory profile, i.e., higher score in those attributes (often) positively associated with consumer preference, e.g., more intense chicken aroma and more intense umami taste. Similarly, FB diets improved some attributes in BG leg samples, though not significantly, when compared to control, e.g., decrease in barn and metallic aroma. In general, the sensory profiles for Bresse Gauloise, especially reared with FB diets, showed a slight improvement in organoleptic properties.

The effect of FB diets in Vorwerkhuhn (VH) led to several organoleptic changes in breast and leg samples, particularly in aroma, flavor, and texture. In the overall profile of breast samples, the effect of FB was reflected by the improvement of some attributes, particularly barn aroma and tenderness. The VC- content led to the least intense barn aroma of all samples. A similar effect was observed in tenderness, as the VC- diet showed the highest tenderness in breast samples. Interestingly, for both attributes, the opposite effect was noticed with a VC+ content diet, suggesting that a faba-bean-based diet only improves the aroma and tenderness when the VC content is low. Our results also show that FB diets favored the flavor of leg samples by reducing the metallic taste and the overall aftertaste when compared to a soy-based diet. The effect of a low-VC-content diet improved the texture of leg samples, particularly their juiciness. Therefore, there appears to be no consistent faba bean effect for VH, but VC content remains important in determining organoleptic quality.

The White Rock (WR) sensory profile deviated from BG and VH, which was not surprising given that WR is a laying breed, whereas the other two are traditional dual-purpose breeds. In breast samples, the FB diets mostly had a negative effect on texture attributes, particularly for tenderness, which is an important attribute associated with overall liking of chicken. In leg samples, the FB diets also affected aroma and flavor attributes although not statistically significantly. These changes were also observed in the other two breeds, where barn aroma and metallic aroma and taste decreased with the inclusion of faba beans in the diet. Similar to breast samples, FB diets had a negative effect on texture attributes, namely firmness and particularly crispiness in WR. While Lawlor et al. [38] showed that firmness is a desired attribute in chicken breast meat, the preference for crispiness is unknown. Nonetheless, it could be accepted that (older) WR animals fed with faba beans will not likely produce the most acceptable poultry meat on the market.

Due to the differing slaughter ages, it is difficult to compare the three different breeds since genotype, age, feed, slaughter conditions, and production systems can influence the sensory profile of chicken breast meat. According to [44], genetic variation only accounts for small differences in taste attributes, whereas age has been shown to increase the intensity of aroma and flavor in meat [4,39]. Our results also show a slight increase in overall flavor for breeds reared longer, especially when comparing the overall flavor of the three breeds fed with control (soy-based) feed. Therefore, the numerical difference amongst the breeds of this study should be interpreted with caution.

Finally, it is important to mention that consumers see the concept of dual-purpose breeds as a more animal-friendly practice [1,5,47,48] for which they would be willing to pay a higher price if meat quality is improved while their expectations on animal welfare are met [1,4,5]. Additionally, studies have shown that consumers are willing to pay more for regional products [49,50]. Consequently, consumers should be willing to pay more for this production system of dual-purpose local breeds fed with regional feedstuff, especially when doing so would improve meat quality parameters.

These results are of relevance to the poultry industry, particularly to breeders of particular local breeds used for dual purposes. This production system offers small-scale breeders an opportunity to target niche markets that demand more ethical or sustainable production methods. The improvement of meat quality parameters and the increase in overall flavor of meat from chickens fed with faba beans show a promising future for this production system.

## 5. Conclusions

Occasionally, differences in meat quality when compared to a status quo soybean meal control group can be attributed to the presence of faba beans or differing vicin and convicin contents in cultivars. However, the effects of diet remain relatively small across all three breeds under investigation, and the presence of faba beans usually improves meat quality parameters. Most interesting is the limited effect that vicin and convicin cultivars have on meat quality, where faba bean diets often present themselves as similar within a breed compared to the soybean meal control. The exception is the VH, where meat quality is slightly negatively impacted by the higher vicin and convicin content but remains on par with the control. Overall, faba beans appear to be an acceptable dietary protein source for rearing local breeds for meat production.

## Figures and Tables

**Table 1:** Ingredient composition of each experimental diet.

	Control	Vicin+	Vicin-
Ingredients (%)			
Wheat	30.0	8.0	8.0
Corn	36.0	25.2	25.2
Soybean meal	24.4	-	-
Blue sweet lupines, cv. Boruta	-	28.6	28.6
Peas, cv. Astronaute	-	10.5	10.5
Faba beans, cv. Fuego	-	20.2	-
Faba beans, cv. Tiffany	-	-	20.2
Grass meal	5.6	0.1	0.1
Soybean oil	0.2	2.7	2.7
Dicalcium phosphate	1.3	2.2	2.2
Calcium carbonate	1.0	0.7	0.7
Salt (NaCl)	0.3	0.4	0.4
DL-Methionine	0.2	0.4	0.4
Vilomix Broiler premix 77047 ^1^	1.0	1.0	1.0
Chemical analyses			
Dry matter (%)	90.0	90.3	90.1
Ash (g/kg DM)	67.6	64.5	64.9
Crude protein (g/kg DM)	211.6	220.5	228.3
Crude fat (g/kg DM)	29.7	56.2	58.7
Crude fiber (g/kg DM)	43.8	60.4	68.5
Methionine (%)	0.49	0.48	0.43
Cysteine (%)	0.30	0.27	0.29
Lysine (%)	0.97	1.01	1.07
Theonine (%)	0.71	0.66	0.69
Vicin (%)	0.005	0.095	0.016
Convicin (%)	0.003	0.043	0.006
VC (Vicin + Convicin; %)	0.008	0.138	0.022
Tannin (mg/g)	4.22	4.48	4.01

^1^ Vitamin–mineral premix provided per kg of diet: Fe, 32 mg; Cu, 12 mg; Zn, 80 mg; Mn, 100 mg; Se, 0.4 mg; I, 1.6 mg; Co, 0.64 mg; retinol, 3.6 mg; cholecalciferol, 0.088 mg; tocopherol, 40 mg; menadione, 4.5 mg; thiamine, 2.5 mg; riboflavin, 8 mg; pyridoxine, 6 mg; cobalamin, 32 µg; nicotinic acid, 45 mg; pantothenic acid, 15 mg; folic acid, 1.2 mg; biotin, 50 µg; choline chloride, 550 mg. Source: Adapted from [23].

**Table 2:** Means (standard deviations) for physicochemical parameters (*n* = 10): pH_20_, pH_24_, pH_72_, color (L* = lightness, a* = redness, b* = yelowness), storage loss, cooking loss, and shear force of breast muscle per breed (BG = Bresse Gauloise, VH = Vorwerkhuhn, WR = White Rock) and feed (C = control, VC+ = high in vicin, VC- = low in vicin).

Breed	BG			VH			WR		
Diet	C	VC+	VC-	C	VC+	VC-	C	VC+	VC-
pH									
pH_20_	6.27 (0.20)	6.29 (0.19)	6.29 (0.29)	6.18 (0.22)	6.41 (0.21)	6.26^1^ (0.16)	6.32 (0.25)	6.26 (0.17)	6.23 (0.23)
pH_24_	5.92 (1.03)	5.67 (0.44)	5.73 (0.45)	5.78 (0.31)	5.75 (0.22)	5.74 (0.30)	5.84 (0.13)	6.09 (0.13)	5.96 (0.35)
pH_72_	5.49 ^a^ (0.33)	5.10 ^b^ (0.16)	5.27 ^a,b^ (0.22)	5.96 (0.18)	5.96^2^ (0.23)	5.86 (0.40)	5.43 ^a^ (0.33)	5.70 ^b^ (0.08)	5.57 ^a,b^ (0.14)
Color with skin									
L*_24_	64.09 (3.04)	61.58 (3.60)	62.89 (3.47)	68.09 (1.90)	67.79 (3.70)	67.38 (4.75)	63.19 (5.24)	65.58 (3.84)	65.71 (2.58)
a*_24_	3.61 (2.02)	3.57 (1.79)	3.13 (0.86)	3.12 (1.49)	3.57 (0.90)	3.46 (1.83)	2.30 (1.09)	1.21 (0.75)	2.05 (1.63)
b*_24_	15.15 (3.53)	15.93 (2.18)	17.53 (1.87)	14.19 (2.30)	15.70 (2.20)	15.88 (2.25)	18.19 ^a^ (4.09)	13.64 ^b^ (2.77)	15.12 ^a,b^ (3.06)
Color without skin									
L*_24_	61.78 (4.44)	60.19 (2.58)	63.24 (2.87)	62.83 (2.18)	61.70 (1.69)	62.32 (2.48)	62.17 ^a,b^ (1.69)	61.43 ^a^ (2.43)	64.03 ^b^ (2.55)
a*_24_	0.10 ^a^ (0.53)	0.83 ^b^ (0.58)	0.58 ^a,b^ (0.45)	0.73 (0.73)	1.33 (0.72)	1.11 (0.67)	0.14 (0.52)	−0.42 (0.47)	0.10 (0.53)
b*_24_	10.64 (2.06)	12.36 (2.65)	12.53 (1.77)	10.05 (2.33)	10.59 (2.01)	9.82 (1.29)	9.76 ^a^ (1.76)	5.98 ^b^ (1.46)	7.61 ^c^ (1.20)
L*_72_	61.20 (4.03)	60.46 (2.38)	62.25 (3.20)	61.05 (2.26)	60.48 (1.92)	61.02 (1.67)	61.05 (1.63)	59.73 (2.52)	61.36 (2.42)
a*_72_	0.88 ^a^ (0.40)	1.54 ^b^ (0.75)	1.23 ^a,b^ (0.51)	1.46 (0.75)	1.94 (0.75)	1.55 (0.61)	1.00 (0.37)	0.90 (0.59)	0.97 (0.56)
b*_72_	10.59 (1.17)	12.46 (2.04)	12.39 (1.80)	9.24 (2.36)	10.25 (1.89)	9.22 (1.31)	8.58 ^a^ (0.93)	7.18 ^b^ (1.03)	8.10 ^a,b^ (1.32)
Water holding capacity									
Storage loss (%)	2.60 (0.34)	2.34 (0.54)	2.21 (0.44)	2.33 (0.22)	2.16 (0.34)	2.01 (0.41)	2.23 ^a^ (0.29)	1.31 ^b^ (0.43)	1.53 ^c^ (0.54)
Cooking loss (%)	21.87 (1.53)	20.79 (1.40)	21.31 (0.97)	20.54 (1.19)	20.21 (1.14)	20.49 (0.89)	21.99 ^a^ (0.98)	22.57 ^a,b^ (1.36)	23.58 ^b^ (1.34)
Instrumental tenderness									
Shear force (N)	3.91 (0.76)	3.82 (0.64)	3.93 (0.91)	4.83 (0.68)	5.21 (0.83)	5.04 (0.99)	5.29 ^a^ (0.76)	4.60 ^b^ (0.36)	4.51 ^c^ (0.63)

^a,b,c^ Values within a breed with differing superscript letters are statistically significantly different (α = 0.05). ^1^ *n* = 8 due to missing measurements at time of observation. ^2^ *n* = 9 due to missing measurements at time of observation.

**Table 3:** Means (standard deviations) for IMP, AMP, and inosine content of breast (*n* = 5) and leg (*n* = 10) muscles per breed (BG = Bresse Gauloise, VH = Vorwerkhuhn, WR = White Rock) and feed (C = control, VC+ = high in vicin, VC- = low in vicin).

Breed	BG			VH			WR		
Diet	C	VC+	V-	C	VC+	V-	C	VC+	V-
Breast (*n =* 5)									
IMP	248 (17)	251 (44)	273 (10)	318 (26)	320 (46)	305 (42)	307 (35)	332 (21)	288 (39)
AMP	4 (1)	3(2)	4 (2)	7 (4)	10 (3)	7 (3)	9 (4)	5 (2)	10 (4)
Inosine	51 (9)	58(9)	60 (8)	17 (3)	17(4)	17(3)	20 (4)	16 (6)	21 (13)
Leg (*n =* 10)									
IMP	147 (13)	141 (11)	146 (14)	147 (12)	150 (11)	143 (12)	151 ^a^ (9)	146 ^a^ (10)	138 ^b^ (11)
AMP	4(2)	2 (1)	2.3 (0.8)	4 (2)	4 (2)	3 (2)	3 (2)	4 (2)	4 (1)
Inosine	11 (3)	12 (3)	10 (3)	8 (3)	8 (2)	8 (2)	6 (2)	6 (2)	6.5 (0.8)

All values are presented in mg/100g. ^a,b,^ Values within a breed with differing superscript letters are statistically significantly different (α = 0.05).

**Table 4:** Estimated marginal means of statistically significant (α = 0.05) sensory attributes (*n* = 10, r = 3), as quantified using unstructured line scales (0 = not perceptible, 100 = strongly perceptible), of chicken breast samples per breed (BG = Bresse Gauloise, VH = Vorwerkhuhn, WR = White Rock) and feed (C = control, VC+ = high in vicin, VC- = low in vicin).

Breed	BG			VH			WR		
Diet	C	VC+	VC-	C	VC+	VC-	C	VC+	VC-
Aroma									
Barn	17.6	18.8	20.7	19.9 ^a,b^	21.5 ^a^	17.4 ^b^	19.7	22.7	19.4
Appearance									
Fibrousness	42.3 ^a,b^	37.8 ^a^	43.2 ^b^	44.9	40.9	43.7	44.6	39.1	41.8
Texture									
Tenderness	71.0	70.1	66.6	61.7 ^a,b^	60.1 ^a^	68.4 ^b^	70.7 ^a^	67.2 ^a,b^	63.6 ^b^

^a,b^ Values within a breed with differing superscript letters are statistically significantly different (α = 0.05).

**Table 5:** Estimated marginal means of statistically significant (α = 0.05) sensory attributes (*n* = 10, r = 2), as quantified using unstructured line scales (0 = not perceptible, 100 = strongly perceptible), of leg samples per breed (BG = Bresse Gauloise, VH = Vorwerkhuhn, WR = White Rock) and feed (C = control, VC+ = high in vicin, VC- = low in vicin).

Breed	BG			VH			WR		
Diet	C	VC+	VC-	C	VC+	VC-	C	VC+	VC-
Aroma									
Barn	19.3	14.3	13.6	10.7 ^a^	14.6 ^b^	12.3 ^a,b^	20.9	16.2	16.4
Flavor									
Greasy/oily	36.3	40.3	41.9	34.8	36.4	36.0	38.9 ^a^	38.0 ^a,b^	33.3 ^b^
Metallic	15.4	13.9	13.8	18.8 ^a^	14.3 ^b^	16.5 ^a,b^	18.3	17.2	15.1
Aftertaste	25.5	24.9	25.3	28.9 ^a^	26.5 ^a,b^	25.3 ^b^	26.4	26.7	24.6
Texture									
Crispiness	21.6 ^a^	32.7 ^b^	35.4 ^b,c^	29.8	28.1	38.6	36.8 ^a^	30.8 ^a,b^	23.9 ^b^
Juiciness	49.1	48.5	50.1	49.3 ^a,b^	43.0 ^a^	51.4 ^b^	46.3	48.3	48.8

^a,b,c^ Values within a breed with differing superscript letters are statistically significantly different (α = 0.05).

## Supplementary Materials

The following are available online at https://www.mdpi.com/2304-8158/9/8/1052/s1, Table S1: Sensory estimated marginal mean attribute scores (standard error) and mixed model statistics (*n* = 10, r = 3) for all Bresse Gauloise breast samples per feed (C = control, VC+ = high in vicin, VC- = low in vicin) group, Table S2: Sensory estimated marginal mean attribute scores (standard error) and mixed model statistics (*n* = 10, r = 3) for all Vorwerkhuhn breast samples per feed (C = control, VC+ = high in vicin, VC- = low in vicin) group, Table S3: Sensory estimated marginal mean attribute scores (standard error) and mixed model statistics (*n* = 10, r = 3) for all White Rock breast samples per feed (C = control, VC+ = high in vicin, VC- = low in vicin) group, Table S4: Sensory estimated marginal mean attribute scores (standard error) and mixed model statistics (*n* = 10, r = 2) for all Bresse Gauloise leg samples per feed (C = control, VC+ = high in vicin, VC- = low in vicin) group, Table S5: Sensory estimated marginal mean attribute scores (standard error) and mixed model statistics (*n* = 10, r = 2) for all Vorwerkhuhn leg samples per feed (C = control, VC+ = high in vicin, VC- = low in vicin) group, Table S6: Sensory estimated marginal mean attribute scores (standard error) and mixed model statistics (*n* = 10, r = 2) for all White Rock leg samples per feed (C = control, VC+ = high in vicin, VC- = low in vicin) group. Raw data for physicochemical parameters and sensory data are also available in supplementary materials.

## Appendix A

### Attribute Definitions and Scales for Breast and Leg Samples

**Table A1:** Sensory attributes, definitions, and scales used to evaluate breast samples.

Attribute	Definition ^1^	Scale
Odor		
Overall intensity	The sum of all perceptible odors.	Not perceptible–Very perceptible
Animal/barn	The intensity of smell of animal/stable.	
Metallic	The intensity of smell of metal/blood.	
Cooked chicken	The intensity of smell of cooked, unseasoned chicken or chicken soup.	
Appearance		
Color intensity	Intensity of the beige color on the cut side.	Light–Dark
Fibrousness	Degree of visible fibers on the cut side of the sample.	Not recognizable–Very recognizable
Moisture release	Amount of moisture that is released after pressing the sample with a fork.	Dry–Moist
Taste		
Overall intensity	The sum of all perceptible flavors.	Not perceptible–Very perceptible
Sweet	The intensity of sweetness.	
Sour	The intensity of sourness.	
Salty	The intensity of saltiness.	
Bitter	The intensity of bitterness.	
Umami	The intensity of umami taste.	
Cooked chicken	The intensity of the taste of cooked, unseasoned chicken or chicken soup.	
Metallic	The intensity of the taste of metal or blood.	
Aftertaste intensity	The intensity of the aftertaste.	
Texture		
Firmness	Force required to bite through the piece with the incisors.	Soft–Firm
Juiciness	Amount of fluid released during the first three chews.	Not juicy–Very juicy
Adhesiveness	Cohesion of the sample during chewing.	Not cohesive–Very cohesive
Tenderness	Force required to chew the piece until it can be swallowed.	Not tender–Very tender
Crumbliness	Number of pieces formed before swallowing; how strongly the mass holds togther or decays during chewing.	Not crumbly–Very crumbly

^1^ Definitions were suggested and accepted by the panelists.

**Table A2:** Sensory attributes, definitions, and scales used to evaluate leg samples.

Attribute	Definition ^1^	Scale
Odor		
Overall intensity	The sum of all perceptible odors.	Not perceptible–Very perceptible
Roasted	The intensity of smell of roasted meat.	
Cooked chicken	The intensity of smell of cooked, unseasoned chicken or chicken soup.	
Animal/barn	The intensity of smell of animal/stable.	
Metallic	The intensity of smell of metal/blood.	
Appearance		
Crust color	The intensity of the brown color of the crust.	Light–Dark
Meat color	The intensity of the brown color of the meat on the cut side of the sample.	Light–Dark
Taste		
Overall intensity	The sum of all perceptible flavors.	Not perceptible–Very perceptible
Sweet	The intensity of sweetness.	
Umami	The intensity of umami taste.	
Cooked chicken	The intensity of the taste of cooked, unseasoned chicken or chicken soup.	
Roasted	The intensity of the taste of roasted (seasoned) meat.	
Fat/oily	The intensity of the taste of fat/oil.	
Metallic	The intensity of the taste of metal or blood.	
Aftertaste intensity	The intensity of the aftertaste.	
Texture		
Crispiness	Force (and intensity of noise) required to break the piece with the incisors.	Not crispy–Very crispy
Firmness	Force required to bite through the piece with the incisors.	Soft–Firm
Juiciness	Amount of fluid released during the first three chews.	Not juicy–Very juicy
Rubbery	Force applied while chewing until the piece can be swallowed.	Not rubbery–Very rubbery
Greasy mouthfeel	Intensity of physical greasy sensation in the mouth caused by fat particles in the sample.	Not greasy–Very greasy

^1^ Definitions were suggested and accepted by the panelists.
