# Supplementary material for: Meat Quality Parameters and Sensory Properties of One High-Performing and Two Local Chicken Breeds Fed with Vicia faba
Source: Foods. 2020 Aug 4;9(8):1052. doi: 10.3390/foods9081052 (PMC7466303; doi:10.3390/foods9081052)
Supplement: Supplementary file 1 [file foods-09-01052-s001.zip › Supplementary files/Supplementary file_Tables.pdf]

Meat quality parameters and sensory properties of two local and one high performing chicken breeds fed with *Vicia faba*.

Cynthia I. Escobedo del Bosque, Brianne A. Altmann, Marco Ciulu, Ingrid Halle, Simon Jansen, Tanja Nolte, Henner Simianer, Steffen Weigend, Daniel Mörlein

Supplementary Materials

Table S1. Sensory estimated marginal mean attribute scores (standard error) and mixed model statistics (n=10, r=3) for all Bresse Gauloise breast samples per feed (C=control, VC+ = high in vicin, VC-= low in vicin) group.

	Feed				Feed effect
Attribute	C	VC+	VC-	SEM	p value
Aroma					
Overall	65.0	64.6	65.2	3.29	0.959
Barn	17.6	18.8	20.7	2.30	0.147
Metallic	11.8	11.5	12.9	1.50	0.454
Chicken	46.9	50.1	51.9	2.56	0.138
Appearance					
Color	19.2	21.7	21.7	2.86	0.198
Fibrousness	42.3^{ab}	37.8^{a}	43.2^{b}	6.63	0.097
Moisture	48.7	53.5	50.7	5.46	0.578
Flavor					
Overall	56.7	57.6	58.8	3.39	0.729
Sweet	17.4	20.9	19.4	2.20	0.213
Sour	27.7	24.1	27.1	3.23	0.597
Salty	16.5	15.6	16.4	2.62	0.724
Bitter	9.7	9.5	10.1	2.62	0.936
Umami	20.5	22.1	25.2	3.32	0.184
Chicken	48.1	48.6	48.2	4.20	0.953
Metallic	15.7	16.6	16.5	2.78	0.739
Aftertaste	28.8	31.8	31.6	3.45	0.362
Texture					
Firmness	40.2	41.1	43.7	5.42	0.466
Juiciness	47.4	50.9	45.4	5.55	0.362
Cohesiveness	47.1	51.1	48.7	6.12	0.602
Tenderness	71.0	70.1	66.6	3.55	0.448
Crumbliness	56.4	58.7	54.7	4.89	0.428

Mixed model: fixed effect feedstuff, random effects panelist, animal, feedstuff*panelist, feedstuff*animal

^{a,b} Means followed by differing superscript letters are statistically significantly different (α=0.05).

Table S2. Sensory estimated marginal mean attribute scores (standard error) and mixed model statistics (n=10, r=3) for all Vorwerkhuhn breast samples per feed (C=control, VC+ = high in vicin, VC-= low in vicin) group.

	Feed				Feed effect
Attribute	C	VC+	VC-	SEM	p value
Aroma					
Overall	62.8	64.2	61.3	4.12	0.430
Barn	20.0^{ab}	21.5^{a}	17.4^{b}	2.35	0.026
Metallic	13.8	12.8	12.3	1.71	0.483
Chicken	49.6	45.5	48.2	3.54	0.623
Appearance					
Color	23.7	23.0	22.4	2.95	0.831
Fibrousness	45.0	40.9	43.7	7.03	0.631
Moisture	49.7	52.9	57.8	6.37	0.404
Flavor					
Overall	58.0	58.8	59.4	3.71	0.918
Sweet	20.7	20.3	19.7	2.05	0.844
Sour	28.9	26.5	28.6	2.66	0.679
Salty	15.9	15.7	17.0	2.72	0.447
Bitter	11.4	9.7	11.1	3.20	0.535
Umami	22.3	24.5	24.1	3.78	0.461
Chicken	46.5	49.6	47.7	4.44	0.264
Metallic	15.3	15.5	17.3	2.66	0.433
Aftertaste	31.8	32.4	32.0	4.30	0.968
Texture					
Firmness	46.8	49.6	48.9	4.96	0.718
Juiciness	48.7	44.5	51.1	4.85	0.273
Cohesiveness	51.0	45.6	52.9	6.57	0.305
Tenderness	61.7^{ab}	60.1^{a}	68.4^{b}	4.27	0.066
Crumbliness	55.2	56.2	60.9	5.02	0.132

Mixed model: fixed effect feedstuff, random effects panelist, animal, feedstuff*panelist, feedstuff*animal ^{a,b} Means followed by differing superscript letters are statistically significantly different (α=0.05).

Table S3. Sensory estimated marginal mean attribute scores (standard error) and mixed model statistics (n=10, r=3) for all White Rock breast samples per feed (C=control, VC+ = high in vicin, VC-= low in vicin) group.

	Feed				Feed effect
Attribute	C	VC+	VC-	SEM	p value
Aroma					
Overall	64.7	65.8	63.9	3.77	0.801
Barn	19.7	22.7	19.4	2.36	0.124
Metallic	12.3	12.7	12.9	1.76	0.898
Chicken	50.2	51.0	52.0	3.48	0.814
Appearance					
Color	20.1	20.9	22.2	2.72	0.461
Fibrousness	44.6	39.1	41.8	6.94	0.143
Moisture	57.1	59.6	57.3	5.60	0.623
Flavor					
Overall	59.5	56.9	55.6	3.37	0.240
Sweet	20.8	18.5	19.2	2.30	0.497

Sour	25.5	27.0	22.7	3.39	0.471
Salty	16.4	16.2	16.3	2.48	0.983
Bitter	10.5	9.7	10.3	2.81	0.869
Umami	21.3	20.9	20.10	2.82	0.744
Chicken	48.3	48.1	46.2	4.12	0.579
Metallic	16.3	15.1	15.1	2.49	0.564
Aftertaste	30.1	30.1	29.2	3.54	0.861
Texture					
Firmness	45.2	48.2	46.5	5.21	0.743
Juiciness	55.5	51.5	49.6	4.77	0.288
Cohesiveness	49.3	51.5	50.0	5.89	0.790
Tenderness	70.7^{a}	67.2^{ab}	63.6^{b}	3.19	0.104
Crumbliness	54.8	51.6	57.8	5.13	0.286

Mixed model: fixed effect feedstuff, random effects panelist, animal, feedstuff*panelist, feedstuff*animal ^{a,b} Means followed by differing superscript letters are statistically significantly different (α=0.05).

Table S4. Sensory estimated marginal mean attribute scores (standard error) and mixed model statistics (n=10, r=2) for all Bresse Gauloise leg samples per feed (C=control, VC+ = high in vicin, VC-= low in vicin) group.

	Feed				Feed effect
Attribute	C	VC+	VC-	SEM	p value
Aroma					
Overall	61.6	61.7	58.5	3.70	0.477
Roasted	43.2	49.4	43.8	4.08	0.160
Chicken	46.6	50.1	47.1	3.67	0.622
Barn	19.3	14.3	13.6	2.82	0.249
Metallic	11.5	11.0	9.6	2.23	0.358
Appearance					
Crust color	32.1	36.3	35.3	5.83	0.398
Meat color	29.6	34.6	34.2	4.77	0.341
Flavor					
Overall	61.2	61.0	60.8	2.52	0.975
Sweet	17.3	17.6	16.9	2.39	0.862
Umami	25.3	27.1	25.9	3.01	0.544
Chicken	58.3	57.2	57.0	2.89	0.761
Roasted	41.9	42.6	42.6	3.72	0.963
Greasy/oily	36.3	40.3	42.0	2.29	0.186
Metallic	15.4	13.9	13.8	2.16	0.666
Aftertaste	25.5	24.9	25.3	3.39	0.942
Texture					
Crispiness	21.6^{a}	32.7^{b}	35.4^{bc}	5.91	0.024
Firmness	34.5	32.9	34.0	5.27	0.886
Juiciness	49.1	48.5	50.1	3.77	0.922
Rubbery	25.9	22.6	22.1	3.18	0.228
Greasy mouthfeel	44.4	45.1	42.2	3.43	0.583

Mixed model: fixed effect feedstuff, random effects panelist, feedstuff*panelist

^{a,b,c} Means followed by differing superscript letters are statistically significantly different (α=0.05).

Table S5. Sensory estimated marginal mean attribute scores (standard error) and mixed model statistics (n=10, r=2) for all Vorwerkhuhn leg samples per feed (C=control, VC+ = high in vicin, VC-= low in vicin) group.

	Feed				Feed effect
Attribute	C	VC+	VC-	SEM	p value
Aroma					
Overall	61.8	61.1	63.6	3.24	0.659
Roasted	51.2	49.9	53.6	3.50	0.482
Chicken	46.0	44.8	47.6	2.92	0.533
Barn	10.7^{a}	14.6^{b}	12.3^{ab}	2.52	0.106
Metallic	10.0	10.0	11.6	2.14	0.137
Appearance					
Crust color	56.9	61.4	59.3	4.69	0.518
Meat color	46.9	44.1	47.9	4.39	0.464
Flavor					
Overall	63.7	60.8	64.8	2.36	0.177
Sweet	15.0	15.3	14.4	1.85	0.898
Umami	27.8	26.2	26.6	3.13	0.716
Chicken	56.1	54.0	56.6	2.24	0.353
Roasted	48.6	45.0	48.9	3.99	0.364
Greasy/oily	34.8	36.4	36.0	2.78	0.853
Metallic	18.8^{a}	14.3^{b}	16.5^{ab}	2.39	0.016
Aftertaste	28.9^{a}	26.5^{ab}	25.3^{b}	3.73	0.119
Texture					
Crispiness	29.8	28.1	38.6	5.69	0.123
Firmness	42.9	50.3	40.2	5.44	0.135
Juiciness	49.3^{ab}	43.0^{a}	51.4^{b}	4.11	0.093
Rubbery	34.2	39.8	32.0	4.71	0.235
Greasy mouthfeel	40.8	40.0	43.1	3.18	0.622

Mixed model: fixed effect feedstuff, random effects panelist, feedstuff*panelist

[a,b,c] Means followed by differing superscript letters are statistically significantly different (α=0.05).

Table S6. Sensory estimated marginal mean attribute scores (standard error) and mixed model statistics (n=10, r=2) for all White Rock leg samples per feed (C=control, VC+ = high in vicin, VC-= low in vicin) group.

	Feed				Feed effect
Attribute	C	VC+	VC-	SEM	p value
Aroma					
Overall	62.9	62.0	58.9	2.83	0.297
Roasted	47.8	48.8	44.1	3.59	0.350
Chicken	46.3	45.3	45.7	3.13	0.903
Barn	20.9	16.2	16.4	2.96	0.329
Metallic	12.0	11.7	11.3	2.77	0.775
Appearance					
Crust color	48.3	43.2	45.6	6.03	0.552
Meat color	39.5	37.5	35.8	4.76	0.568
Flavor					
Overall	61.2	59.1	60.5	2.58	0.674
Sweet	14.4	15.9	16.0	2.12	0.548

Umami	27.3	24.5	26.0	2.43	0.343
Chicken	56.1	52.6	56.2	2.49	0.141
Roasted	46.3	42.8	41.8	3.90	0.186
Greasy/oily	38.9[a]	38.0[ab]	33.3[b]	1.97	0.075
Metallic	18.3	17.2	15.1	2.60	0.164
Aftertaste	26.4	26.7	24.6	3.36	0.456
Texture					
Crispiness	36.8[a]	30.8[ab]	24.0[b]	5.47	0.044
Firmness	46.9	41.4	47.5	5.17	0.238
Juiciness	46.3	48.3	48.8	3.56	0.644
Rubbery	37.5	34.2	38.7	4.39	0.300
Greasy mouthfeel	41.0	43.7	39.5	3.55	0.296

Mixed model: fixed effect feedstuff, random effects panelist, feedstuff*panelist

[a,b,c] Means followed by differing superscript letters are statistically significantly different (α=0.05).
